# Mycobacterial Interspersed Repetitive Unit Can Predict Drug Resistance of *Mycobacterium tuberculosis* in China

**DOI:** 10.3389/fmicb.2016.00378

**Published:** 2016-03-23

**Authors:** Xian-feng Cheng, Chao Jiang, Min Zhang, Dan Xia, Li-li Chu, Yu-feng Wen, Ming Zhu, Yue-gen Jiang

**Affiliations:** ^1^School of Public Health, Wannan Medical CollegeWuhu, China; ^2^Clinical Laboratory, Institute of Dermatology, Chinese Academy of Medical Sciences – Peking Union Medical CollegeNanjing, China; ^3^Clinical Laboratory, Zhongda Hospital, School of Medicine, Southeast UniversityNanjing, China; ^4^Pediatric Research Institute, Nanjing Children’s Hospital, Nanjing Medical UniversityNanjing, China; ^5^Clinical Laboratory, Ma’anshan Center for Disease Control and PreventionMa’anshan, China

**Keywords:** *Mycobacterium tuberculosis*, Mycobacterial Interspersed Repetitive Unit, drug resistance, repeat units, genotyping

## Abstract

**Background:** Recently, Mycobacterial Interspersed Repetitive Unit (MIRU) was supposed to be associated with drug resistance in *Mycobacterium tuberculosis* (*M. tuberculosis*), but whether the association exists actually in local strains in China was still unknown. This research was conducted to explore that association and the predictability of MIRU to drug resistance of Tuberculosis (TB).

**Methods:** The clinical isolates were collected and the susceptibility test were conducted with Lowenstein–Jensen (LJ) medium for five anti-TB drug. Based on PCR of MIRU-VNTR (Variable Number of Tandem Repeat) genotyping, we tested the number of the repeat unite of MIRU. Then, we used logistic regression to evaluate the association between 15 MIRU and drug resistance. In addition, we explored the most suitable MIRU locus of identified MIRU loci for drug resistance by multivariate logistic regression.

**Results:** Of the 102 strains, one isolate was resistant to rifampicin and one isolate was resistant to streptomycin. Among these fifteen MIRU, there was a association between MIRU loci polymorphism and anti-tuberculosis drug resistance, ETRB (*P* = 0.03, *OR* = 0.19, 95% CI 0.05–0.81) and ETRC (*P* = 0.01, OR = 0.14, 95% CI 0.03–0.64) were negatively related to isoniazid resistance; MIRU20 (*P* = 0.05, *OR* = 2.87, 95% CI 1.01–8.12) was positively associated with ethambutol resistance; and QUB11a (*P* = 0.02, *OR* = 0.79, 95% CI 0.65–0.96) was a negative association factor of p-aminosalicylic acid resistance.

**Conclusion:** Our research showed that MIRU loci may predict drug resistance of tuberculosis in China. However, the mechanism still needs further exploration.

## Introduction

Tuberculosis (TB) caused by *Mycobacterium tuberculosis* (*M. tuberculosis*) infection has affected human beings for 1000s of years. Due to patients defaulting on TB therapy, there has been an increasing number of drug resistant TB strains, especially multidrug-resistant TB (MDR-TB; Biadglegne et al., 2014). In 2012, 1.3 million people died from TB, of which 3.6% of all new and 20% of all recurring cases involved infection with MDR-TB^1^. Particularly, the prevalent genotype in East Asia, Beijing genotype *M. tuberculosis*, has shown drug resistance (Liu et al., 2012). Drug resistance, especially MDR-TB, has made treating TB extremely difficult.

Genetic factors play important roles in drug resistance specific to TB treatment. For example, isoniazid (INH), rifampicin (RFP), and streptomycin (SM) resistance were found to be related to genes katG315 (Lu et al., 2014), rpoB513 (Jun et al., 2009), and gidB A80P (Perdigão et al., 2014), respectively. In addition, an eﬄux pump capable of eﬄuxion of INH was found to be related to five genes (Rodrigues et al., 2012). The underlying mechanisms of these resistances, however, are still unclear.

Mycobacterial Interspersed Repetitive Unit is a type of the “minisatellite” VNTR (Variable Number of Tandem Repeat) with the length of MIRU repetitive unit ranging from 50 to 100 base pairs (bp) (Supply et al., 2001). A series of studies suggested that MIRU loci could be involved in the expression regulation of neighboring genes. For example, when the repeat units of Mtub39 locus repeat fourth, the expression level of its downstream gene *lpdA* was 12-fold as that of the same gene when the frequency of repeat units of Mtub39 locus was 1 (Akhtar et al., 2009). Similar results were showed in another experiment (Pérez-Lago et al., 2013): ∼56% increase in expression level of *lpdA* when the frequency of repeat units of Mtub39 was from 3 to 4. Tantivitayakul et al. (2010) also found that MIRU10 could promote the expression of downstream gene *gfp*. In addition, different frequencies of tandem repeats may influence the folding of DNA, thereby affecting the attraction, binding, and interaction of transcription factors (Supply et al., 1997).

Our previous studies have confirmed/verified the hypothesis that some MIRU loci polymorphism were related to anti-tuberculosis drug resistance (Yu-feng et al., 2015). This study was conducted using strains worldwidely, of which 54 were from Germany, 20 were from Ghana, 20 were from Uganda, and 15 were from former Soviet Union. However, whether the association between MIRU loci and drug resistance exists in China or other countries were still unknown. Therefore, in this study, we sampled 102 clinical strains of *M. tuberculosis* from eastern China to explorethe association between the MIRU loci and drug resistance.

## Materials and Methods

### Selection of Clinical Isolates

A total of 102 clinical isolates of *M. tuberculosis* were selected from sputum of patients who were recruited between 2009 and 2012 in the eastern area of Anhui Province, China. The standard *M. tuberculosis* strain H37Rv was used as the control isolate, which was courteously provided by Dr. Chuanyou Li of Beijing Chest Hospital, China.

### Drug Susceptibility Testing

Four first-line anti-tuberculosis drugs including RFP, INH, SM, ethambutol (EMB), and one second-line drug of p-aminosalicylic acid (PAS) were incorporated into LJ medium, at the following concentrations: INH, 0.2 μg/mL; RFP, 40.0 μg/mL; SM, 4.0 μg/mL; EMB, 2.0 μg/mL; PAS, 1.0 μg/mL^2^. The proportion method was used to detect the drug-resistance of the *M. tuberculosis*. Strain was scored as resistant to a specific drug, or defined as sensitive if its growth rate was <1% compared to the control. Strain isolation, identification, and drug susceptibility tests (DST) were performed at the laboratory of Ma Anshan Centre for Disease Control and Prevention.

### MIRU Loci Selection

According to the Chinese genotyping handbook and protocol referenced literature (Lee et al., 2014), 15 MIRU loci were selected in this study. The primer sequence and length of repeat units of each MIRU locus was shown in **Table 1**.

**Table 1:** The promoter sequence and the length of repeat units of 15 MIRU loci.

MIRU locus	Promoter sequence(5′–3′)	Length of repeat units (bp)
ETRC	F:GTGAGTCGCTGCAGAACCTGCAG R:GGCGTCTTGACCTCCACGAGTG	45
ETR-D	F:CAGGTCACAACGAGAGGAAGAGC R:GCGGATCGGCCAGCGACTCCTC	45
ETR-E	F:CTTCGGCGTCGAAGAGAGCCTC R:CGGAACGCTGGTCACCACCTAAG	45
QUB11a	F:CCCATCCCGCTTAGCACATTCGTA R:TTCAGGGGGGATCCGGGA	42
QUB11b	F:CGTAAGGGGGATGCGGGAAATAGG R:CGAAGTGAATGGTGGCAT	42
MIRU-10	F:GTTCTTGACCAACTGCAGTCGTCC R:GCCACCTTGGTGATCAGCTACCT	47
MIRU-16	F:TCGGTGATCGGGTCCAGTCCAAGTA R:CCCGTCGTGCAGCCCTGGTAC	46
MIRU-20	F:TCGGAGAGATGCCCTTCGAGTTAG R:GGAGACCGCGACCAGGTACTTGTA	48
MIRU-26	F:CCCGCCTTCGAAACGTCGCT R:TGGACATAGGCGACCAGGCGAATA	44
MIRU-27	F:TCGAAAGCCTCTGCGTGCCAGTAA R:GCGATGTGAGCGTGCCACTCAA	46
MTUB-31	F:GTGACGTTTACCGTGCTCTATTTC R:GTCGTCGGACAGTTCTAGCTTT	46
MIRU-39	F:CGCATCGACAAACTGGAGCCAAAC R:CGGAAACGTCTACGCCCCACACAT	48
MIRU-40	F:AAGCGCAAGAGCACCAAG R:GTGGGCTTGTACTTGCGAAT	38
ETRA	F:AAATCGGTCCCATCACCTTCTTAT R:CGAAGCCTGGGGTGCCCGCGATTT	48
ETR-B	F:GCGAACACCAGGACAGCATCATG R:GGCATGCCGGTGATCGAGTGG	44

### DNA Preparation and PCR Amplification

Sputum specimens were treated with 4% NaOH and inoculated on LJ medium for 4–6 weeks. The colony of 1–2 rings was dissolved in 400 μL sterile distilled water on the vortex device. In addition, the liquid supernatant was stored with -20°C cryopreservation after being boiled in a water bath for 30 min and clarified by centrifugation at 4°C at 12,000 rpm for 15 min.

Each PCR mix contained 12.5 μL of 2x PCR buffer (Tiangen Biotech Co., Ltd., Beijing, China), 0.25 μM of each primer and 6 μL of sterile distilled water. Four microliters of template DNA was added to each reaction mixture. Following an initial denaturation at 95°C for 10 min the PCRs were subjected to 40 thermocycles of 94°C for 60 s, 60°C for 60 s, and 72°C for 90 s. This was followed by a final extension at 72°C for 10 min. Positive and negative control reactions, in which PCR mixes were inoculated with 4 μL of boiled cells from the *M. tuberculosis* H37RV strain and sterile distilled water, respectively, were performed with each set of reactions (Lee et al., 2014).

The PCR products were electrophoretically separated on 2% agarose gel (Life Technologies, Grand Island, NY, USA) in 1x Tris-boric acid-EDTA buffer (Tiangen). A 100-bp DNA stepladder (Thermo Fisher Scientific Inc., Waltham, MA, USA) was used as the marker.

### Data Analysis

The allelic diversity (*h*-value) of MIRU loci, individually and in combination, was calculated by the following equation: $h=1-\Sigma x_{i}^{2}\left[n/\left(n-1\right)\right]$, where *n* is the number of isolates and *x*_i_ is the frequency of its allele at the locus (Kremer et al., 2005).

Univariate logistic regression with the enter method was used to initially evaluate the associations between MIRU loci and anti-tuberculosis drug resistance. Multivariate logistic regression with the stepwise method was used to analyze the major risk and protective factors of MIRU loci initially selected from the univariate logistic regression.

## Results

### Distribution of Repeat Units, Polymorphism, and Drug Resistance Rate of MIRU Loci

The number of repeat units in the 15 MIRU loci ranged from 0 to 12. The *h*-value, which measures the allelic diversity of MIRU loci, ranged from 0.03 for ETRC to 0.77 for OUB11a. Of the drug resistance, 22 were resistant to INH, 20 were resistant to RFP, 5 were resistant to EMB, and 26 were resistant to PAS. The anti-TB drug rates of tuberculosis strains with different copies of MIRU locus unit ranged from 0.0 to 100.0% (Supplementary Table 1).

### Association between MIRU Loci and Drug-Resistance in *M. tuberculosis*

According to univariate logistic regression analysis, we found a number of MIRU loci associated with the drug resistance (**Table 2**). For example, ETRB was associated with INH resistance (*P* < 0.005). The strain with one repeat of ETRB is the most drug resistant: 6 out of 10 are INH resistant, versus 16 out of 87 being resistant for strains with two repeats of ETRB (Supplementary Table 1). The corresponding odds ratio (OR) was estimated to be 0.14. Overall, 3, 4, and 3 out of the 15 MIRU loci showed resistance to INH, EMB, PAZ, respectively. No MIRU loci were associated with RFP and SM resistance.

**Table 2:** Association between the MIRU loci and drug resistance in univariate logistic regression.

Model	Loci	β	*SE*	*P*	OR	95% CI for OR	
7-8						Lower limit	Upper limit
INH	MIRU40	–0.88	0.43	0.04	0.41	0.18	0.97
	ETRB	–1.97	0.68	<0.005	0.14	0.04	0.53
	ETRC	–2.26	0.80	<0.005	0.10	0.02	0.50
EMB	MIRU20	1.05	0.53	0.048	2.87	1.01	8.12
	MIRU27	–1.27	0.60	0.03	0.28	0.09	0.91
	MIRU40	–1.26	0.63	0.047	0.29	0.08	0.98
	ETRC	–1.45	0.63	0.02	0.23	0.07	0.80
PAS	ETRA	–0.96	0.42	0.02	0.38	0.17	0.87
	ETRB	–1.31	0.63	0.04	0.27	0.08	0.92
	QUB11a	–0.24	0.10	0.02	0.79	0.65	0.96

In addition, the ability of the MIRU loci in predicting drug resistance was studied by multivariate logistic regression analysis. Overall, three models were found to provide predictions. For example, with MIRU40, ETRB, and ETRC were the most suitable MIRU loci for the predictive model. We also found other models being able to improve prediction for EMB and PAS resistance (**Table 3**). The models detected here could potentially aid in future drug resistance prediction.

**Table 3:** Association between the MIRU loci and drug resistance in multivariate logistic regression.

Model	Loci	β	*SE*	*P*	OR	95% CI for OR	
7-8						Lower limit	Upper limit
INH	ETRB	–1.66	0.74	0.03	0.19	0.05	0.81
	ETRC	–1.98	0.79	0.01	0.14	0.03	0.64
	Constant	7.53	2.62	0.00			
EMB	MIRU20	1.05	0.53	0.05	2.87	1.01	8.12
	Constant	–5.83	1.58	0.00			
PZA	QUB11a	–0.24	0.10	0.02	0.79	0.65	0.96
	Constant	0.38	0.63	0.54			

## Discussion

In the present study, we found that ETRB and ETRC were the major predicted factors for INH resistance and QUB11a was the leading predicted factor for PAS resistance. The allelic diversity of QUB11a (0.77) was high but that of ETRB (0.25) and ETRC (0.17) was low.

Although no functions have been found for many MIRU loci, there are evidences that certain MIRU loci can affect nearby gene activities. For example, Yao et al. found that Mtub-39 could affect the promotor activity of the downstream genes, and subsequently regulate the expression of the genes^3^. Along with our previous results (Wen et al., 2015), the identified resistance-related MIRU in our study provide good hints for generating function-related hypotheses for these MIRU loci, which was briefly described in the following paragraphs. Particularly, MIRU loci might exert functions by regulating the expression of neighboring genes.

For INH resistance, ETRB was immediately downstream of gene *qcrB*. It has been reported that overexpression of *qcrB* in *Mycobacterium bovis* bacillus Calmette–Guérin (BCG) enabled the bacteria to survive at imidazo[1,2-a]pyridines(IP) concentrations that would normally be lethal (Abrahams et al., 2012). Cytochrome b encoded by *qcrB* was the subunit of bc_1_ compound which formed a key component in the electron transport chain and included in superoxide generation (Crofts et al., 2008). Previous studies have found that repeated ALU sequences can depress the expression of the reporter gene *GFP* (Li et al., 2012). We hypothesized that increased copy numbers of ETRB units might inhibit the expression of *qcrB*, and lead to sensitivity to INH, whose anti-tuberculosis function is associated with the electron transport chain (Herman and Weber, 1980; Lee et al., 2013). ETRC, another protective factor for INH resistance, is located 210 bp upstream of *Rv0488*.

For EMB resistance, MIRU20 locus is a protective factor. The frequency of MIRU20, located 19 bp upstream of *Rv1817*, is 2 in H37Rv strain. The function of *Rv1817* is still unknown, and is believed to be probably involved in cellular metabolism and metal utilization (Camacho et al., 1999). Interestingly, Zhang et al. (2013) reported that the mutation of *Rv1817* may be associated with ofloxacin and kanamycin resistance but not EMB. Thus, we hypothesized that MIRU20 may stimulate the expression of *Rv1817*, and result in the EMB resistance. However, potential molecular mechanisms about *Rv1817* involved in EMB resistance have not been found previously.

QUB11a, a major protective factor for PAS resistance, is included in the gene *PPE34*. Expression of a recombinant *PPE34* protein in *Mycobacterium smegmatis* showed that *PPE34* was likely to be both exposed on the surface of the cell and associated with the cell wall (Lu et al., 2009). *PPE34* is polymorphic and related to changes in the numbers of tandem repeat. The high number of copy units of QUB11a may influence the activity of the expression protein of gene PPE34 and then impair the barrier function of cell wall to have *M. tuberculosis* be resistant to PAS.

In this study, we found that MIRU 40 was related to the increased INH resistance, which was the same as that in our previous results (Wen et al., 2015).

## Conclusion

Our research showed that MIRU loci may predict drug resistance of tuberculosis in China. However, the mechanism and exact predictive model still need further exploration.

At last, a number of limitations exist in our study. First, only 15 MIRU loci of *M. tuberculosis* were selected. This is partially because our work was designed based on the tuberculosis genotyping guideline of Chinese version as the strains were all from China and we thought the initial research method would be better according to the handbook of China version. Second, there is still lack of direct evidence for the mechanisms mentioned above. Further functional study is needed to clarify whether the abnormal expression of neighboring genes can cause resistance. Third, the study may need to include other MIRU loci to investigate the association of MIRU loci with drug resistance in *M. tuberculosis*.

## Author Contributions

X-fC, M. Zhang, M. Zhu, and Y-gJ: acquisition, analysis, and interpretation of data for the work. CJ, DX, and L-lC: drafting the work or revising it critically for important intellectual content. Y-fW: substantial contributions to the conception or design of the work. Final approval of the version to be published.

## Conflict of Interest Statement

The authors declare that the research was conducted in the absence of any commercial or financial relationships that could be construed as a potential conflict of interest.

## Footnotes

1 http://www.who.int/tb/publications/global_report/en/

2 http://www.cdc.gov/tb/topic/laboratory/Drug_testing.htm

3 http://www.stmopen.net/research-on-relationship-between-polymorphisms-of-mtub-39-and-expression-of-downstream-genes-of-mycobacterium-tuberculosis/
