# Supplementary material for: Mycobacterial Interspersed Repetitive Unit Can Predict Drug Resistance of Mycobacterium tuberculosis in China
Source: Front Microbiol. 2016 Mar 23;7:378. doi: 10.3389/fmicb.2016.00378 (PMC4803746; doi:10.3389/fmicb.2016.00378)
Supplement: Supplementary file 1 [file Table_1.DOCX]

**Supplement Table 1. The distribution of repeat units, polymorphism and drug resistance frequency of the MIRU loci**

| MIRU | Repeat | n | *h*-value | INH resistance | RFP resistance | SM resistance | EMB resistance | PSA resistance |
| --- | --- | --- | --- | --- | --- | --- | --- | --- |
| MIRU10 | 1 | 4 | 0.43 | 50.0(2/4) | 0.0(0/4) | 25.0(1/4) | 0.0(0/4) | 0.0(0/4) |
|  | 2 | 74 |  | 21.6(16/74) | 21.6(16/74) | 17.6(13/74) | 5.4(4/74) | 24.3(18/74) |
|  | 3 | 18 |  | 16.7(3/18) | 16.7(3/18) | 11.1(2/18) | 0.0(0/18) | 33.3(6/18) |
|  | 4 | 2 |  | 50.0(1/2) | 50.0(1/2) | 50.0(1/2) | 50.0(1/2) | 50.0(1/2) |
|  | 5 | 4 |  | 0.0(0/4) | 0.0(0/4) | 0.0(0/4) | 0.0(0/4) | 25.0(1/4) |
| MIRU16 | 0 | 1 | 0.54 | 100.0(1/1) | 0.0(0/1) | 100.0(1/1) | 0.0(0/1) | 0.0(0/1) |
|  | 1 | 6 |  | 50.0(3/6) | 33.3(2/6) | 33.3(2/6) | 16.7(1/6) | 66.7(4/6) |
|  | 2 | 33 |  | 15.2(5/33) | 21.2(7/33) | 3.0(1/33) | 3.0(1/33) | 24.2(8/33) |
|  | 3 | 60 |  | 21.7(13/60) | 18.3(11/60) | 21.7(13/60) | 5.0(3/60) | 23.3(14/60) |
|  | 4 | 2 |  | 0.0(0/2) | 0.0(0/2) | 0.0(0/2) | 0.0(0/2) | 0.0(0/2) |
| MIRU20 | 1 | 4 | 0.54 | 25.0(1/4) | 0.0(0/4) | 25.0(1/4) | 0.0(0/4) | 50.0(2/4) |
|  | 2 | 52 |  | 21.2(11/52) | 17.3(9/52) | 9.6(5/52) | 3.8(2/52) | 23.1(12/52) |
|  | 3 | 45 |  | 22.2(10/45) | 22.2(10/45) | 22.2(10/45) | 4.4(2/45) | 24.4(11/45) |
|  | 7 | 1 |  | 0.0(0/1) | 100.0(1/1) | 100.0(1/1) | 100.0(1/1) | 100.0(1/1) |
| MIRU26 | 1 | 4 | 0.56 | 25.0(1/4) | 25.0(1/4) | 0.0(0/4) | 0.0(0/4) | 50.0(2/4) |
|  | 3 | 5 |  | 20.0(1/5) | 0.0(0/5) | 20.0(1/5) | 0.0(0/5) | 0.0(0/5) |
|  | 4 | 3 |  | 33.3(1/3) | 0.0(0/3) | 0.0(0/3) | 0.0(0/3) | 66.7(2/3) |
|  | 5 | 14 |  | 35.7(5/14) | 28.6(4/14) | 14.3(2/14) | 14.3(2/14) | 21.4(3/14) |
|  | 6 | 9 |  | 11.1(1/9) | 22.2(2/9) | 11.1(1/9) | 0.0(0/9) | 44.4(4/9) |
|  | 7 | 65 |  | 16.9(11/65) | 18.5(12/65) | 16.9(11/65) | 3.1(2/65) | 21.5(14/65) |
|  | 8 | 1 |  | 100.0(1/1) | 0.0(0/1) | 100.0(1/1) | 0.0(0/1) | 0.0(0/1) |
|  | 9 | 1 |  | 100.0(1/1) | 100.0(1/1) | 100.0(1/1) | 100.0(1/1) | 100.0(1/1) |
| MIRU27 | 1 | 7 | 0.46 | 28.6(2/7) | 14.3(1/7) | 14.3(1/7) | 28.6(2/7) | 42.9(3/7) |
|  | 2 | 24 |  | 29.2(7/24) | 16.7(4/24) | 16.7(4/24) | 4.2(1/24) | 29.2(7/24) |
|  | 3 | 70 |  | 18.6(13/70) | 21.4(15/70) | 17.1(12/70) | 2.9(2/70) | 22.9(16/70) |
|  | 4 | 1 |  | 0.0(0/1) | 0.0(0/1) | 0.0(0/1) | 0.0(0/1) | 0.0(0/1) |
| MTUB31 | 2 | 1 | 0.03 | 100.0(1/1) | 100.0(1/1) | 0.0(0/1) | 100.0(1/1) | 0.0(0/1) |
|  | 3 | 100 |  | 21.0(21/100) | 19.0(19/100) | 17.0(17/100) | 4.0(4/100) | 26.0(26/100) |
|  | 12 | 1 |  | 0.0(0/1) | 0.0(0/1) | 0.0(0/1) | 0.0(0/1) | 0.0(0/1) |
| MTUB39 | 1 | 16 | 0.53 | 37.5(6/16) | 12.5(2/16) | 18.8(3/16) | 12.5(2/16) | 43.8(7/16) |
|  | 2 | 19 |  | 21.1(4/19) | 21.1(4/19) | 10.5(2/19) | 0.0(0/19) | 21.1(4/19) |
|  | 3 | 65 |  | 18.5(12/65) | 21.5(14/65) | 18.5(12/65) | 4.6(3/65) | 23.1(15/65) |
|  | 4 | 2 |  | 0.0(0/2) | 0.0(0/2) | 0.0(0/2) | 0.0(0/2) | 0.0(0/2) |
| MIRU40 | 1 | 3 | 0.33 | 66.7(2/3) | 33.3(1/3) | 33.3(1/3) | 33.3(1/3) | 66.7(2/3) |
|  | 2 | 11 |  | 45.5(5/11) | 18.2(2/11) | 18.2(2/11) | 9.1(1/11) | 27.3(3/11) |
|  | 3 | 82 |  | 15.9(13/82) | 18.3(15/82) | 15.9(13/82) | 3.7(3/82) | 23.2(19/82) |
|  | 4 | 4 |  | 50.0(2/4) | 50.0(2/4) | 25.0(1/4) | 0.0(0/4) | 50.0(2/4) |
|  | 5 | 2 |  | 0.0(0/2) | 0.0(0/2) | 0.0(0/2) | 0.0(0/2) | 0.0(0/2) |
| ETRA | 2 | 4 | 0.31 | 50.0(2/4) | 50.0(2/4) | 25.0(1/4) | 0.0(0/4) | 50.0(2/4) |
|  | 3 | 12 |  | 33.3(4/12) | 25.0(3/12) | 33.3(4/12) | 25.0(3/12) | 50.0(6/12) |
|  | 4 | 83 |  | 19.3(16/83) | 18.1(15/83) | 14.5(12/83) | 2.4(2/83) | 21.7(18/83) |
|  | 5 | 3 |  | 0.0(0/3) | 0.0(0/3) | 0.0(0/3) | 0.0(0/3) | 0.0(0/3) |
| ETRB | 1 | 10 | 0.25 | 60.0(6/10) | 40.0(4/10) | 30.0(3/10) | 10.0(1/10) | 50.0(5/10) |
|  | 2 | 87 |  | 18.4(16/87) | 18.4(16/87) | 16.1(14/87) | 4.6(4/87) | 24.1(21/87) |
|  | 3 | 4 |  | 0.0(0/4) | 0.0(0/4) | 0.0(0/4) | 0.0(0/4) | 0.0(0/4) |
|  | 5 | 1 |  | 0.0(0/1) | 0.0(0/1) | 0.0(0/1) | 0.0(0/1) | 0.0(0/1) |
| ETRC | 1 | 3 | 0.17 | 100.0(3/3) | 33.3(1/3) | 33.3(1/3) | 33.3(1/3) | 66.7(2/3) |
|  | 2 | 5 |  | 60.0(3/5) | 20.0(1/5) | 20.0(1/5) | 20.0(1/5) | 40.0(2/5) |
|  | 3 | 92 |  | 17.4(16/92) | 19.6(18/92) | 16.3(15/92) | 3.3(3/92) | 23.9(22/92) |
|  | 4 | 1 |  | 0.0(0/1) | 0.0(0/1) | 0.0(0/1) | 0.0(0/1) | 0.0(0/1) |
|  | 5 | 1 |  | 0.0(0/1) | 0.0(0/1) | 0.0(0/1) | 0.0(0/1) | 0.0(0/1) |
| ETRD | 1 | 1 | 0.26 | 0.0(0/1) | 0.0(0/1) | 0.0(0/1) | 0.0(0/1) | 0.0(0/1) |
|  | 2 | 8 |  | 62.5(5/8) | 25.0(2/8) | 37.5(3/8) | 25.0(2/8) | 37.5(3/8) |
|  | 3 | 87 |  | 17.2(15/87) | 19.5(17/87) | 16.1(14/87) | 3.4(3/87) | 23.0(20/87) |
|  | 4 | 5 |  | 20.0(1/5) | 20.0(1/5) | 0.0(0/5) | 0.0(0/5) | 40.0(2/5) |
|  | 5 | 1 |  | 100.0(1/1) | 0.0(0/1) | 0.0(0/1) | 0.0(0/1) | 100.0(0/1) |
| ETRE | 2 | 3 | 0.43 | 33.3(1/3) | 0.0(0/3) | 33.3(0/3) | 0.0(0/3) | 33.3(1/3) |
|  | 3 | 12 |  | 33.3(4/12) | 16.7(2/12) | 8.3(1/12) | 0.0(0/12) | 33.3(4/12) |
|  | 4 | 7 |  | 42.9(3/7) | 28.6(2/7) | 28.6(2/7) | 28.6(2/7) | 42.9(3/7) |
|  | 5 | 75 |  | 18.7(14/75) | 20.0(15/75) | 17.3(13/75) | 4.0(3/75) | 24.0(18/75) |
|  | 6 | 2 |  | 0.0(0/2) | 0.0(0/2) | 0.0(0/2) | 0.0(0/2) | 0.0(0/2) |
|  | 7 | 1 |  | 0.0(0/1) | 0.0(0/1) | 0.0(0/1) | 0.0(0/1) | 0.0(0/1) |
|  | 8 | 1 |  | 0.0(0/1) | 0.0(0/1) | 0.0(0/1) | 0.0(0/1) | 0.0(0/1) |
|  | 10 | 1 |  | 0.0(0/1) | 100.0(0/1) | 0.0(0/1) | 0.0(0/1) | 0.0(0/1) |
| QUB11a | 1 | 8 | 0.77 | 50.0(4/8) | 37.5(3/8) | 37.5(3/8) | 25.0(2/8) | 50.0(4/8) |
|  | 2 | 2 |  | 0.0(0/2) | 0.0(0/2) | 0.0(0/2) | 0.0(0/2) | 50.0(1/2) |
|  | 3 | 2 |  | 0.0(0/2) | 0.0(0/2) | 0.0(0/2) | 0.0(0/2) | 100.0(2/2) |
|  | 4 | 4 |  | 25.0(1/4) | 0.0(0/4) | 25.0(1/4) | 0.0(0/4) | 50.0(2/4) |
|  | 5 | 13 |  | 23.1(3/13) | 23.1(3/13) | 7.7(1/13) | 0.0(0/13) | 23.1(3/13) |
|  | 6 | 20 |  | 15.0(3/20) | 20.0(4/20) | 5.0(1/20) | 10.0(2/20) | 15.0(3/20) |
|  | 7 | 6 |  | 33.3(2/6) | 33.3(2/6) | 16.7(1/6) | 0.0(0/6) | 16.7(1/6) |
|  | 8 | 40 |  | 20.0(8/40) | 17.5(7/40) | 22.5(9/40) | 2.5(1/40) | 25.0(10/40) |
|  | 9 | 5 |  | 20.0(1/5) | 20.0(1/5) | 20.0(1/5) | 0.0(0/5) | 0.0(0/5) |
|  | 10 | 2 |  | 0.0(0/2) | 0.0(0/2) | 0.0(0/2) | 0.0(0/2) | 0.0(0/2) |
| QUB11b | 1 | 3 | 0.56 | 0.0(0/3) | 0.0(0/3) | 0.0(0/3) | 0.0(0/3) | 33.3(1/3) |
|  | 2 | 1 |  | 100.0(1/1) | 0.0(0/1) | 0.0(0/1) | 0.0(0/1) | 100.0(1/1) |
|  | 3 | 8 |  | 25.0(2/8) | 37.5(3/8) | 12.5(1/8) | 0.0(0/8) | 37.5(3/8) |
|  | 4 | 6 |  | 50.0(3/6) | 33.3(2/6) | 33.3(2/6) | 0.0(0/6) | 33.3(2/6) |
|  | 5 | 14 |  | 21.4(3/14) | 7.1(1/14) | 7.1(1/14) | 14.3(2/14) | 21.4(3/14) |
|  | 6 | 65 |  | 20.0(13/65) | 21.5(14/65) | 20.0(13/65) | 4.6(3/65) | 24.6(16/65) |
|  | 7 | 2 |  | 0.0(0/2) | 0.0(0/2) | 0.0(0/2) | 0.0(0/2) | 0.0(0/2) |
|  | 8 | 3 |  | 0.0(0/3) | 0.0(0/3) | 0.0(0/3) | 0.0(0/3) | 0.0(0/3) |
